# Meddling with middle modalities: a decomposition approach to mental health inequalities between intersectional gender and economic middle groups in northern Sweden

**DOI:** 10.3402/gha.v9.32819

**Published:** 2016-11-24

**Authors:** Per E. Gustafsson, Miguel San Sebastián, Paola A. Mosquera

**Affiliations:** Epidemiology and Global Health, Department of Public Health and Clinical Medicine, Umeå University, Umeå, Sweden

**Keywords:** intersectionality, socioeconomic factors, health inequality, mental health, gender, Sweden, decomposition analysis

## Abstract

**Background:**

Intersectionality has received increased interest within population health research in recent years, as a concept and framework to understand entangled dimensions of health inequalities, such as gender and socioeconomic inequalities in health. However, little attention has been paid to the intersectional middle groups, referring to those occupying positions of mixed advantage and disadvantage.

**Objective:**

This article aimed to 1) examine mental health inequalities between intersectional groups reflecting structural *positions* of gender and economic affluence and 2) decompose any observed health inequalities, among middle groups, into contributions from experiences and conditions representing *processes* of privilege and oppression.

**Design:**

Participants (*N*=25,585) came from the cross-sectional ‘Health on Equal Terms’ survey covering 16- to 84-year-olds in the four northernmost counties of Sweden. Six intersectional positions were constructed from gender (woman vs. men) and tertiles (low vs. medium vs. high) of disposable income. Mental health was measured through the General Health Questionnaire-12. Explanatory variables covered areas of material conditions, job relations, violence, domestic burden, and healthcare contacts. Analysis of variance (Aim 1) and Blinder-Oaxaca decomposition analysis (Aim 2) were used.

**Results:**

Significant mental health inequalities were found between dominant (high-income women and middle-income men) and subordinate (middle-income women and low-income men) middle groups. The health inequalities between adjacent middle groups were mostly explained by violence (mid-income women vs. men comparison); material conditions (mid- vs. low-income men comparison); and material needs, job relations, and unmet medical needs (high- vs. mid-income women comparison).

**Conclusions:**

The study suggests complex processes whereby dominant middle groups in the intersectional space of economic affluence and gender can leverage strategic resources to gain mental health advantage relative to subordinate middle groups.

## Introduction

Social inequalities in health are a pressing concern for the Swedish society, and socioeconomically disadvantaged women seem to fare the worst when it comes to health (1). Research on health inequalities has, however, been criticized for failing to capture such intersecting inequalities, and for concentrating on demonstrating the existence of health inequalities rather than studying the underlying processes (2). This study uses an intersectional approach as the basis for decomposing intersecting gender and economic inequalities in mental health in northern Sweden.

The concept of intersectionality, developed by the legal scholar Kimberlé Crenshaw (3, 4), has gained increased attention within population health research as a theoretical framework of multiple intertwined axes of inequality (2, 5–7). The adoption of intersectionality by population health research has not been frictionless, however, and a particular issue of contention has been how to translate intersectionality theory into quantitative methodology (2, 5, 8). It has further been argued that an intersectional approach for population health should distinguish between *social positions* – which comprise potentials for privilege, oppression, and marginalization on the one hand – and *social processes*, which (re)produce or counteract health inequalities across social positions on the other; and ideally both positions and processes should be examined (2). The reflection of intersectional positions in population patterns of health can here be understood as a process of embodiment (9, 10), whereby social inequalities, through pathways of embodiment, become expressed in individual bodies and thereby create health inequalities.

Intersectionality research within population health has so far mostly focused on the overall pattern of health outcomes across contrasting intersectional positions (11, 12) or emphasized the ‘extreme groups’ of multiple disadvantage or advantage, for example, high-income men or low-income women (5, 13). A range of measures capturing the consequences of intersectional positions have also been formulated, for example, see the review by Jackson et al. (14). Less attention has been paid to the health consequences of people belonging to mixed locations (2, 8, 15), that is, people who are occupying positions of mixed advantage or disadvantage, for example, financially well-off women or low-income men.

As argued by Sen and Iyer (15), such intersectional ‘middle groups’ differ from the ‘extreme groups’ in ways which may be particularly illuminating to understand the processes of intersectionality. They, furthermore, describe how multiple middle groups can be understood as comprising *dominant* and *subordinate* middle groups (15). Here, dominant middle groups are those that have a structural advantage along one axis relative to a subordinate middle group, such as middle-income men having a structural gender advantage over middle-income women, as well as an economic advantage over low-income men (15). Middle groups are structurally adjacent, and either dominant or subordinate, to at least one other middle group, and they may therefore be involved in the direct struggle for resources and entitlements in different arenas, such as at home, in the public space, and at work. In contrast to the extreme groups, holding a mixed position of simultaneous advantage and disadvantage also enables *leveraging* of structural advantages along one axis to counteract the disadvantages along another axis, in order to gain a secondary advantage. Moreover, although any social and health inequalities between intersectional middle groups are expected to be smaller than between extreme groups, the middle groups commonly represent a considerable portion of the population. Sen et al. (15, 16) have proposed a methodological approach to highlight intersectional middle groups, and have illustrated how leveraging is used between economic and gender middle groups to secure entitlements to health care in India (15). In this study, leveraging is understood as processes whereby middle groups can gain relative advantages in terms of mental health, and leveraging can therefore be construed as a pathway of embodiment.

The specific arrangements of a society's welfare systems, health system, and gender order influence the possibilities of effective leveraging. Sweden enjoys a comparatively gender and economic equitable society (17), with universal health care, well-developed social protections systems, and progressive gender equality policies, for example, with respect to parental leave (18). These contextual characteristics of Sweden would be expected to translate into relatively small gender and economic inequalities in health, and little room for intersectional middle groups to leverage a relative advantage to counteract a relative disadvantage. Nevertheless, processes of oppression, marginalization, and discrimination are still operating in daily life across multiple spheres in Sweden. For example, despite the social welfare systems in place, economical resources are still strongly shaping the mental health of Swedish women and men (19). Moreover, despite high labor market participation for women in Sweden, the gendered division of labor is expressed in women taking an undue share of unpaid domestic work (20). Both women and those of socioeconomic disadvantage may also be engaged in jobs characterized by a poorer psychosocial work environment (21) and less secure contracts (22), compared with men and those of socioeconomic advantage. Exposure to gender violence is another extreme expression of gender inequalities that can impact mental health (23), and other forms of degrading or humiliating treatment in everyday life have also shown to be important for health and healthcare seeking in both Swedish women and men, but particularly among those socioeconomically disadvantaged (24, 25). Further processes of marginalization involve discrimination in the health system based on gender and/or socioeconomic disadvantage (25, 26), or refraining from seeking necessary health care due to shortage of funds (27). As such, there are multiple processes which may be relevant for leveraging between middle groups of economic and gender intersections in Sweden, and which thereby can act as pathways of embodiment upholding mental health inequalities.

This article aims to employ the approach suggested by Sen and Iyer (15) by 1) examining mental health inequalities between intersectional groups reflecting structural *positions* of gender and economic affluence; and to develop the approach by 2) decomposing any observed health inequalities between dominant and subordinate middle groups to experiences and conditions representing processes of privilege and oppression, using Blinder-Oaxaca decomposition analysis.

## Methods

### Study population and procedures

The study population comprised the participants of the cross-sectional population-based survey ‘Health on Equal Terms’ carried out in spring 2014 by the County Councils of the four northernmost counties of Sweden: Norrbotten, Västerbotten, Jämtland/Härjedalen, and Västernorrland. The target population included all residents in the four counties aged 16–84 years. The sample frame consisted of 704,099 individuals of the target population, identified through the Total Population Register of Statistics Sweden on November 30, 2013. Sampling was done in two steps. First, as part of the national ‘Health on Equal Terms’ survey, a small national sample (*N*=1,789; 3.4%) was randomly selected without stratification. Second, as part of the regional expanded sample, a larger regional random sample (*N*=50,300; 96.6%) stratified into 276 strata by county, municipality, gender, and age was selected (28, 29). The overall participation rate was 49%, resulting in a sample size of *N*=25,667.

No additional inclusion or exclusion criteria were applied to the study, and all participants with valid responses on gender and income were eligible for inclusion in the analysis. In total, 25,585 individuals were included: 14,988 of which belonged to middle groups (see Measures, Exposure: intersectional positions by gender and income, below) and therefore included for the multiple analyses. Due to item non-response, effective *N* was 24,580–25,585 in descriptive/bivariate analyses on the total sample, and *N*=13,385 in multiple analyses on the subsample comprising the middle groups.

The survey was implemented through postal questionnaire covering areas of health and well-being, drug use and health care contacts, health behaviors, and working and social conditions. In addition, sociodemographic individual-level data, such as annual income (for 2012) and country of birth, were retrieved from the Total Population Register of Statistics Sweden and linked to the survey data through the unique Swedish Personal Identity Number.

The use of the ‘Health on Equal Terms’ survey in this study was reviewed and approved by the Regional Ethical Review Board in Umeå (approval no. 2015/134–31Ö).

### Measures

#### Outcome: mental health

Mental health symptoms were self-reported through the General Health Questionnaire (GHQ)-12 (30), a commonly used screening instrument for mental health. The GHQ-12 has displayed good psychometric properties in the Swedish setting, including good internal consistency and excellent validity when it comes to detecting depressive disorders (31). The GHQ-12 consists of 12 items covering symptoms during the previous weeks, including lack of concentration, sleeplessness, and moodiness. The items were coded on a four-level Likert scale (1–4). The Likert scores were averaged across the 12 items and multiplied by 12 in order to construct a summative index while avoiding bias due to item non-response. The final index thus had a theoretical range of 36, and sample Cronbach's alpha was 0.89, which is similar to the Swedish validation study (31).

#### Exposure: intersectional positions by gender and income

Gender and income were based on information from population registers of Statistics Sweden. Gender comprised the categories woman and man. Income was measured as annual disposable income for the respondent, which was divided into tertiles (cut-offs at 144,718 and 232,065 SEK) in order to form three equally sized categories of economic affluence: low, medium, and high.

In accordance with the procedure by Sen and Iyer (15), gender and affluence were combined to form six mutually exclusive intersectional positions or groups, including the *extreme groups* of low-income women and high-income men; the *dominant middle groups* of high-income women and medium-income men; and the *subordinate middle groups* of medium-income women and low-income men.

#### Explanatory factors: processes of privilege and oppression

Explanatory variables were selected to capture processes of privilege, oppression, or marginalization relevant for economic affluence and gender, and which potentially could be used as leverage points to gain mental health advantages. An overview of all variables is shown in Table 1.

**Table 1:** Descriptive statistics of all variables in the total sample and by intersectional positions of gender and affluence.

		Women			Men		
			
Variable	Total	Low	Mid	High	Low	Mid	High
*N*	25,585	5,456	4,940	3,387	3,072	3,589	5,141
Depressive symptoms, *M* (SD)	21.4 (4.7)	22.2 (5.1)	21.6 (4.9)	21.2 (4.6)	21.6 (4.9)	21.2 (4.3)	20.4 (3.8)
Demographic factors							
Age							
Young adult: 16–35 years	22.9	37.1	20.7	10.3	41.5	15.0	12.5
Middle age: 36–64 years	44.3	17.4	51.4	76.1	20.5	33.0	66.9
Old age: 66–85 years	32.9	45.5	27.9	13.6	38.0	52.0	20.6
Born outside Sweden	6.3	8.9	6.2	5.0	9.7	4.8	3.6
Education							
Low	50.1	66.6	43.8	25.2	68.4	55.8	42.0
Medium	33.3	25.8	35.9	35.4	26.6	34.6	39.6
High	16.6	7.6	20.3	39.4	5.0	9.6	18.4
Material conditions							
Diff. to make ends meet							
Sometimes	4.9	6.0	5.7	3.7	6.9	4.9	2.8
Often	5.9	8.8	6.4	3.9	9.8	4.8	2.2
Low cash margin	16.4	28.5	16.2	8.6	29.2	11.8	4.9
Residential ownership							
Resident-owned	74.8	60.6	78.3	86.4	57.2	76.1	88.1
Rental	18.2	26.3	18.5	11.9	23.2	19.4	10.1
Other arrangements	7.0	13.2	3.3	1.8	19.6	4.5	1.8
Job relations							
Job dissatisfaction	7.2	5.5	8.3	6.9	7.4	7.7	7.8
Job insecurity	7.7	7.5	9.5	7.6	7.3	6.1	7.4
Violence							
Fear of violence	13.2	25.8	18.6	18.1	5.6	3.9	2.5
Threat/violence experience	4.2	4.5	4.3	4.7	6.2	3.5	2.9
Degrading treatment	16.4	20.5	19.9	19.3	16.8	11.6	9.8
Domestic burden							
Elderly care	9.7	7.6	10.8	13.7	7.7	8.5	10.1
Child illness	3.7	1.9	5.0	7.4	1.3	1.8	4.6
Healthcare contacts							
Unmet medical needs							
Inaccessibility	5.0	6.9	4.7	3.3	6.0	4.8	4.1
Negative experiences	5.6	7.4	6.3	4.3	4.9	5.9	4.1
Other reasons	8.7	11.7	8.7	7.5	10.2	8.3	5.9
Unmet dental care needs							
Economic reasons	7.3	8.9	7.7	4.7	11.1	8.2	4.2
Other reasons	9.1	11.0	8.1	7.7	10.3	10.1	7.8

Numbers are column percentages within each variable, unless otherwise noted.

Material conditions were measured by three variables: *Low cash margin* (whether the respondent would be able to get hold of 15,000 SEK [approx. 1,600 EUR] in 1 week) was coded as ‘no’ (1) and ‘yes’ (0); *Difficulties to make ends meet* (whether the respondent has had difficulties paying running costs during the past 12 months) was coded as ‘no’ (0), ‘yes, once’ (1), and ‘yes, multiple times’ (2); and *Residential ownership* was coded as owned house or apartment (0), rental apartment (1), and other living arrangements (2).

Job relations were measured by two variables: *Job dissatisfaction* (‘How well do you enjoy your work tasks?’) was coded as satisfied (0) and dissatisfied (1); and *Job insecurity* (‘Are you worried about losing your job within the coming year?’) was coded as ‘no’ (0) and ‘yes’ (1).

Violence was measured by three variables: *Fear of violence* (‘Do you ever refrain from walking out alone in fear of being assaulted, robbed, or harassed in any other way?’) was coded as ‘no’ (0) or ‘yes’ (1). *Threat/violence* was based on two items on whether the respondent during the past 12 months had been exposed to physical violence or threat of violence, which were combined into one variable coded as ‘no’ (0) and ‘yes’ (1). *Degrading treatment* was based on whether the respondent during the past 12 months had been treated in a way that was perceived as degrading or humiliating, which was coded as ‘no’ (0) or ‘yes’ (1).

Domestic burden was measured through *Child with illness* (having a child with chronic disease or functional limitation), coded as ‘no’ (0) and ‘yes’ (1); and *Elderly care* (whether the respondent takes care of everyday tasks for someone close who is ill or old), coded as ‘no’ (0) and ‘yes’ (1).

*Healthcare contacts* were measured by five variables concerning unmet medical and dental care needs in the past 3 months, and the reasons for refraining from seeking care. Three variables concerned unmet medical care needs: *Inaccessibility* (including ‘too long waiting times’, ‘difficulties to reach the provider by phone’, ‘too late appointment’, and ‘not knowing where to turn’), *Negative experiences*, and *Other reasons*. Two variables concerned unmet dental care needs, due to *Financial reasons* and *Other reasons*. All variables were coded as ‘no’ (0), including people without perceived healthcare needs and those who sought care when in need, and ‘yes’ (1), including people who refrained from seeking care when in need, that is, with unmet care needs.

#### Demographic background variables

Demographic background was measured by three variables: *Age* was coded as 16–35 years (0), 36–65 years (1), and 66–85 years (2); *Country of birth* was coded as Sweden (0) and outside Sweden (1); and *Education* was coded as low (0), medium (1), and high (2).

### Data analysis

#### Aim 1 analyses

Following the principal approach by Sen et al. (15, 16) but adapted for continuous outcomes, the first aim of illustrating mental health inequalities between intersectional positions was addressed by reporting GHQ-12 means and confidence intervals for all six groups. Absolute mean differences in GHQ-12 score between the groups were subsequently tested through a one-way analysis of variance (ANOVA), with post-hoc tests for all pairwise comparisons using the Games-Howell correction method. Analyses were done using SPSS v23.

#### Aim 2 analyses

The second aim, seeking to explain any health gaps between dominant and subordinate middle groups displayed in the Aim 1 analyses, was addressed by Blinder-Oaxaca decomposition analysis (32) using the *oaxaca* command (33) in Stata v13. The basic idea of Blinder-Oaxaca decomposition is to explain the distribution of the outcome, in this case group difference in mean GHQ-12 score between dominant and subordinate middle groups, by a set of explanatory factors that vary systematically across the groups. The method is based on two linear regression models that are fit separately for each of the groups, and the technique then partitions the health gap between the groups into a fraction attributable to differences in the explanatory factors (the explained part) and to differences in coefficients (the unexplained part; 32).

Blinder-Oaxaca decomposition was applied to decompose absolute difference in mean GHQ-12 score for the following three comparisons between dominant versus subordinate middle groups: 1) middle-income men (dominant) versus middle-income women (subordinate); 2) middle-income men (dominant) versus low-income men (subordinate); and 3) high-income women (dominant) versus middle-income women (subordinate). All explanatory factors described above were included to explain the health gap. The total explained and unexplained parts, as well as the independent contribution of each of the explanatory factor, are reported as absolute contributions (i.e. on the same scale as the outcome) and as relative contributions (percentages). Relative contributions are calculated with respect to the absolute health gap for the total explained and unexplained parts, and relative to the absolute explained part for the individual contributions of each explanatory factor.

## Results

### Descriptive statistics of explanatory factors across intersectional groups

To illustrate the variations in explanatory variables between the different intersectional positions, Table 1 shows descriptive statistics for the total sample as well as for the six intersectional positions. As expected, the doubly disadvantaged group of low-income women generally reported the least favorable life conditions, whereas the doubly advantaged group of high-income men reported the most favorable circumstances. The distributions were more mixed for the four middle groups. The most striking finding was that the economic gradient in fear of violence, present in both women and men, was on a level 5–7 times more frequent in women than in men. Similarly, whereas there was an economic gradient in degrading treatment in men, the gradient was much weaker in women, but at a considerably higher level.

### Mental health inequalities between intersectional groups (Aim 1)

**Fig. 1:**
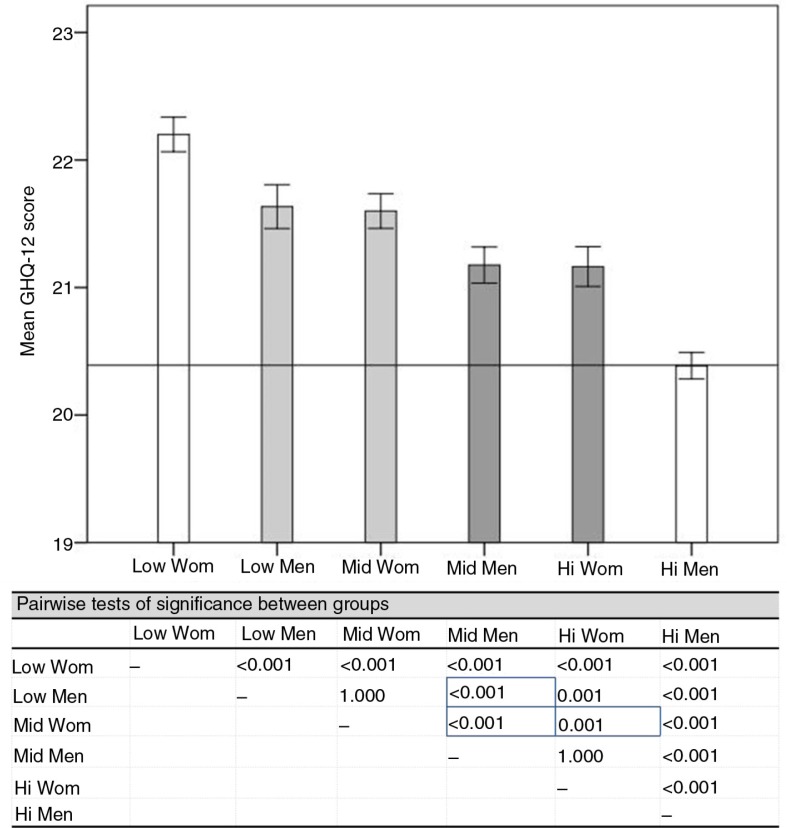
Mental health (mean GHQ-12 score) in intersections by affluence (low, middle, and high income) and gender (woman, man): illustration of means with extreme (white), dominant (dark grey), and subordinate (light grey) groups; and tests of significance between groups (middle groups’ comparisons with border). *p*-values are derived from one-way ANOVA [F(5, 25563)=88.81, *p*<0.001] using Games–Howell post-hoc tests for multiple comparisons.

See Fig. 1 for a display of mean mental health for the six intersectional positions, corresponding to Aim 1. The bar graph shows the intersections ordered by mental health from worst to best, with statistical inference of absolute difference in means from a one-way ANOVA [F(5, 25563) =88.81, *p*<0.001] and *p*-values for all pairwise comparisons in the lower part of the figure. At the extremes are the doubly disadvantaged and advantaged groups of low-income women and high-income men, who reported worst and best health, respectively (mean difference (95% CI)=1.81 (1.57–2.06); *p*<0.001). The health inequalities between the middle groups were as expected of smaller magnitude but were still significant. The subordinate mid-income women reported worse mental health than the dominant mid-income men (0.42 (0.14–0.71); *p*<0.001) and high-income women (0.44 (0.13–0.74); *p*<0.001), and the subordinate low-income men reported worse mental health than the dominant mid-income men (0.46 (0.13–0.78); *p*<0.001). In contrast, very similar mean health was reported by the two subordinate (0.03 (−0.35−0.28); *p*=1.000) and the two dominant (0.01 (−0.32−0.29); *p*=1.000) middle groups. As such, mental health inequalities were observed across the range of extreme, subordinate, and dominant middle groups of gender and affluence.

### Decomposition of mental health gaps between intersectional middle groups (Aim 2)

Table 2 displays a summary of Blinder-Oaxaca decompositions of the mental health gap between structurally adjacent dominant and subordinate middle groups, corresponding to Aim 2. The models explained 68.2% of the gap between mid-income women and mid-income men; 75.3% of the low-income men versus mid-income men gap; and merely 33.5% of the mid-income women versus high-income women gap.

**Table 2:** Summary of Blinder-Oaxaca decomposition analyses of mental health (GHQ-12 score) between intersectional middle groups by affluence and gender

	Mid-income women (group 1) Mid-income men (group 2)			Low-income men (group 1) Mid-income men (group 2)			Mid-income women (group 1) High-income women (group 2)		
			
Model estimates	Abs.	Rel. (%)	*p*	Abs.	Rel. (%)	*p*	Abs.	Rel. (%)	*p*
GHQ-12 mean (group 1)	21.57		0.000	21.64		0.000	21.57		0.000
GHQ-12 mean (group 2)	21.11		0.000	21.11		0.000	21.16		0.000
Health gap	0.469		0.000	0.534		0.000	0.412		0.000
Explained fraction	0.320	68.2	0.000	0.402	75.3	0.000	0.138	33.5	0.023
Unexplained fraction	0.149	31.8	0.161	0.132	24.7	0.296	0.274	66.5	0.010
Factor contributions									
Country of birth	−0.002	−0.7	0.488	0.008	1.9	0.384	−0.004	−2.9	0.212
Age	−0.074	−23.1	0.004	−0.160	−39.7	0.000	−0.026	−18.7	0.342
Education	0.010	3.0	0.568	0.038	9.4	0.022	−0.073	−52.8	0.003
Diff. make ends meet	0.041	12.7	0.001	0.071	17.6	0.002	0.059	42.6	0.000
Low cash margin	0.023	7.2	0.017	0.148	36.8	0.000	0.037	26.5	0.021
Residential ownership	−0.015	−4.7	0.037	0.095	23.6	0.008	0.015	10.9	0.226
Job dissatisfaction	0.011	3.5	0.384	−0.008	−2.0	0.491	0.040	29.0	0.024
Job insecurity	0.027	8.4	0.003	0.017	4.3	0.038	0.018	12.9	0.012
Fear of violence	0.097	30.3	0.000	0.025	6.2	0.023	0.001	0.8	0.778
Threat/violence experience	0.011	3.4	0.098	0.017	4.3	0.058	−0.001	−0.9	0.773
Degrading treatment	0.163	51.0	0.000	0.081	20.2	0.000	0.014	10.5	0.429
Elderly care	0.002	0.6	0.636	0.000	0.0	0.900	−0.011	−8.1	0.045
Child illness	0.022	6.9	0.038	0.002	0.5	0.525	−0.019	−13.6	0.010
Medical: inaccessibility	−0.002	−0.7	0.756	0.015	3.9	0.076	0.023	16.6	0.016
Medical: neg. experiences	0.008	2.4	0.276	−0.010	−2.5	0.306	0.022	15.7	0.006
Medical: other	0.008	2.5	0.443	0.039	9.6	0.006	0.020	14.5	0.111
Dental: other	−0.004	−1.3	0.294	0.002	0.4	0.499	0.001	0.4	0.653
Dental: economic reasons	−0.004	−1.2	0.416	0.023	5.6	0.028	0.023	16.8	0.012

Estimates are absolute (Abs.) and relative (Rel.) contributions and *p*-values.

The better mental health among mid-income men compared with mid-income women was mostly explained by experiences of degrading treatment (alone standing for half of the explained portion of the health gap) and fear of violence in public space (30% of the explained gap). These two factors were also much more frequent in women than in men, across the economic spectrum (Table 1). Difficulties to make ends meet, low cash margin, and job insecurity made smaller but still significant (*p*<0.05) and sizable (7–13%) contributions to the health gap.

In contrast, material conditions were the most important in explaining the mental health advantage of middle-income relative to low-income men. Here, low cash margin, residential ownership, and difficulties to make ends meet jointly explained almost 80% of the explained health gap. Again, this is mirrored by the description in Table 1, where low cash margin and frequent difficulties to make ends meet were 2–3 times more common in low-income than middle-income men. Moreover, experience of degrading treatment made a considerable contribution (20%), with fear of violence and unmet medical and dental needs also making smaller contributions (5–10% each).

The explained portion of the mental health gap between mid- and high-income women was, as noted above, smaller than for the other comparisons, which was due to education statistically offsetting the health gap in the model, as indicated by a large negative contribution (−53%). This offsetting contribution of education appeared due to high-income women having higher education than middle-income women (Table 1), in combination with high education being associated to worse, not better, mental health among mid- to high-income women (data not shown). The most important factors explaining the gap were more spread across different spheres than in the two comparisons mentioned above. Material conditions, including difficulties to make ends meet and low cash margin together explained 69%; job relations, including dissatisfaction and insecurity explaining 42%; and unmet medical needs due to inaccessibility and previous negative experiences jointly explained 32% of the total explained part of the health gap. However, degrading treatment was of less importance, mirroring the similar frequencies of this experience in high- and middle-income women (Table 1).

## Discussion

The present study from northern Sweden first found patterns of mental health across intersections of gender and affluence marked by both *inequalities* – with dominant middle groups reporting better mental health than subordinate middle groups – and *equalities* – with the two dominant middle groups reporting similar mental health, as did the two subordinate middle groups. Second, the observed mental health inequalities between dominant and subordinate middle groups were explained partly by processes of specific importance for each comparison and partly by processes of more universal importance.

There is considerable evidence on mental health differentials between genders and socioeconomic groups (34, 35), including in Sweden (19, 36, 37). In this study, the overall population pattern of mental health across dominant and subordinate middle groups highlights the value of considering middle groups in intersectionality research. For example, despite the structural distinctiveness of the positions of high-income women versus middle-income men and low-income men versus mid-income women, these pairs reported close to identical mental health. These similarities in mental health and the overall small mental health inequalities found in this study should be interpreted in the context of the welfare systems of Sweden, which are expected to reduce health inequalities and also the possibilities for leveraging between groups.

Previous research has attributed gender inequalities in health to, for example, working conditions (21), domestic violence (23), and also to the unequal distribution of sociodemographic factors (38). Economic inequalities in health have similarly been attributed to, for example, material factors such as financial strain and employment conditions (36, 39, 40). This study illustrates how social inequalities underlie the health inequalities between intersectional middle groups. Although the design and analysis do not allow for causal inference, these findings may reflect processes of leveraging, and how gender and economic intersections become embodied, and health inequalities are upheld. As such, the findings could be viewed as suggesting that middle-income men are able to successfully leverage their structural advantages into a mental health advantage relative to both middle-income women and low-income men, but by different processes. The gender advantage indicated by feeling safe in the public space and infrequent experiences of degrading treatment stood out as valuable resources underlying the mental health advantage relative to similarly affluent women. In contrast, economic advantages as indicated by being able to pay for running costs, financial security, and privileged residential conditions seemed to be valuable points of leverage to gain a mental health advantage relative to low-income men. In addition, degrading treatment and making ends meet emerged as important factors to explain the health inequalities among all middle group comparisons. These factors have been emphasized as critical social determinants of health in Swedish women and men (19, 24), which can reflect their usefulness for both gender and economic leveraging.

For the inequality between mid- and high-income women, the health gap was slightly smaller than the economic comparison between middle- and low-income men, and was not explained as well to the same degree as the other comparisons. These findings bear some resemblance with the findings by Griffin (41) from the United Kingdom, where GHQ scores were not different across social grades in women, but in men. Similar to middle-income men, the economic advantage of high-income women was reflected in material advantages, as well as in better work conditions and access to health care. However, high-income women experienced fear of violence and degrading treatment as frequently as did their middle-income counterparts, and these factors also made less contributions to the mental health gap than for the comparison of middle- and low-income men. This suggests that in contrast to men, economic advantage may not be a very effective leverage point to gain a secure life for women, due to the pervasive power of gender disadvantage.

### Methodological considerations

The methodological strengths of the study include a large population-based sample, with a well-validated outcome measure, and utilization of novel statistical approaches. However, the cross-sectional nature of the data precludes any causal inferences. Since only half of those invited participated in the survey, selection bias might have been introduced into the sample. For example, people with severe mental disorders such as major depression could be expected to be under-represented in the sample. This would lead to biased estimates of, for example, mean population mental health, but unless the non-participants simultaneously differ from the sample with respect to, for example, the gender or income, the point estimates of the main analyses would not be expected to be severely biased. Nevertheless, the impact of selection bias is ultimately unknown.

The mental health outcome, GHQ-12, has performed well in the Swedish validation study (31), but is still a screening instrument which potentially can involve inaccurate responses. The use of self-reports can also introduce common-method bias, although in this study at least the main attributes of exposure (gender and income) were measured through independent registers and not self-reported. Moreover, social categories are an issue of contention within intersectionality research, and even pragmatic provisional use of conventional social categories (8) has received criticism (42). Moreover, the sole focus on the affluence–gender intersection naturally disguises within category heterogeneity along, for example, ethnicity or sexuality.

Additional factors not covered by the questionnaire could also be of interest, for example, gender equality at home (20, 43), job strain (37), and discrimination (44). Labor market position was an additional factor which we preliminarily included in the analysis but was highly collinear with age due to the age-diverse sample. As such, the estimate of age can be viewed as capturing both biological age, as well as the age-related labor market circumstances.

## Conclusions

This study gives some indications as to how dominant middle groups in the intersectional space of economic affluence and gender can leverage strategic resources tied to their structural position, in order to gain a mental health advantage relative to subordinate middle groups. From a population health perspective, this highlights how complex pathways of embodiment of entangled gender and economic inequalities may contribute to population patterns of mental health. Future intersectional research should pay attention to intersectional middle groups, and the complex processes of leveraging underlying health inequalities between them. Offering a safe public space, combatting discrimination, and improving financial security are three areas which stand out as promising for policy and prevention seeking to improve gender and economic equity in mental health.

## Paper context

Intersectional population health research has focused mostly on multiple advantaged or disadvantaged groups, but less on the middle groups of simultaneous advantage and disadvantage. This northern Swedish study of mental health inequalities between middle groups at intersections of gender and income indicates how the inequalities are explained by partly general and partly unique circumstances. Policies aiming toward gender and economic equity in mental health ought to consider the complex processes between intersectional middle groups.
